# Editorial: Advances in nutritional strategies for optimizing swine growth performance and gut health

**DOI:** 10.3389/fvets.2026.1910884

**Published:** 2026-07-01

**Authors:** Young Dal Jang, Jae Cheol Jang, Jinsu Hong

**Affiliations:** 1 Department of Animal and Dairy Science, University of Georgia, Athens, GA, United States; 2 Department of Animal Science, Gyeongsang National University, Jinju, Republic of Korea; 3 Department of Animal Sciences, Purdue University, West Lafayette, IN, United States

**Keywords:** growth, gut health, nutrition, pig, sow, weaning

## Introduction

Pre- and post-weaning mortality and morbidity remain major constraints in global swine production, driving substantial economic losses and welfare concerns. These challenges arise from the convergence of immature digestive and immune systems in neonatal piglets, increased litter size and birth weight variability from hyperprolific sow genetics, and the ongoing restriction of in-feed antibiotics and pharmacological zinc oxide. As a result, swine nutrition is increasingly shifting toward functional, biologically targeted strategies that prioritize gut integrity, immune modulation, and microbial stability alongside growth performance.

Modern nutritional approaches now emphasize integrated interventions applied either directly during the nursery phase or indirectly through maternal dietary programming. Across the studies compiled in this Research Topic, a consistent theme emerges: gut health and performance are most effectively improved through multi-mechanistic strategies that simultaneously target nutrient digestion, microbial ecology, epithelial barrier function, and host immunity.

## Nursery-phase functional nutrition: enzymes, fermentation metabolites, and feed additives

A group of studies highlights the central role of digestive optimization strategies, particularly enzyme-based interventions and fermentation-related metabolites. de Medeiros et al. and Atoo et al. collectively demonstrate that exogenous enzyme supplementation such as phytase, xylanase, carbohydrases, protease, alone or combined with acidifiers can improve nutrient digestibility and intestinal health, even under nutritionally challenging conditions such as reduced-protein diets or high soybean meal inclusion. These interventions consistently reduced biomarkers of intestinal irritation, improved amino acid utilization, and mitigated post-weaning diarrhea, supporting their viability as foundational strategies in antibiotic-free production systems. Similarly, da Silva et al. demonstrated the spray-dried plasma inclusion in nursery diet could reduce prevalence of severe postweaning diarrhea, while Benavides-Infante et al. showed that branched-chain volatile fatty acids enhance microbial fermentation efficiency and nutrient utilization, particularly under high-fiber feeding conditions, reinforcing the importance of modulating hindgut fermentation as part of modern feed formulation strategies.

## Microbiome-targeted and barrier-supportive interventions

Several studies converge on strategies aimed at stabilizing gut microbiota and reinforcing epithelial barrier integrity. Guantario et al. demonstrated that prebiotic galacto-oligosaccharides reduce ETEC adhesion and invasion while preserving epithelial integrity and enhancing the growth of probiotic bacteria. Chong et al. extended this concept by showing that hyodeoxycholic acid acts as a microbiota-responsive bile acid modulator, strengthening tight junction expression and promoting *Lactobacillus* enrichment through bile acid signaling pathways. Complementing these findings, Koo et al. reported that *Bacillus*-based biotics and enzyme supplementation improved immune competence and reduced pathogenic bacterial loads, particularly *Mycoplasma* spp. Together, these studies emphasize that microbiome modulation is most effective when it simultaneously supports barrier function, microbial competition, and immune signaling rather than acting through single-target mechanisms.

## Disease resilience, mineral metabolism, and environmental interactions

Additional studies highlight the importance of host resilience mechanisms and the interaction between nutrition and environmental stressors. Tian et al. demonstrated that intramuscular iron injection enhances intestinal barrier function and reduces inflammatory damage during ETEC challenge, reinforcing the role of micronutrient status in infection resilience. Ng'ang'a et al. similarly showed that zinc oxide remains effective in improving metabolic adaptation, immune regulation, and intestinal integrity, although its regulatory phase-out underscores the urgency of identifying functional alternatives.

Importantly, Brandão Melo et al. showed that sanitary conditions and pathogen exposure exert a stronger influence on gut microbial composition than dietary amino acid supplementation, highlighting that environmental stressors may override nutritional modulation under disease pressure. Likewise, Ikeda et al. reported that propolis and probiotic supplementation altered microbial populations but also indicated that crude propolis does neither improve early growth nor reduce post-weaning diarrhea, suggesting that refinement of bioactive compounds is necessary for consistent performance benefits.

## Maternal nutritional programming and transgenerational effects

A second major theme across the Research Topic is the role of maternal nutrition in shaping offspring health and performance. Che et al. demonstrated that maternal supplementation with 3-hydroxyisobutyric acid reduces within-litter birth weight variation and improves neonatal energy availability through enhanced placental lipid transport. Similarly, Xiao et al. showed that maternal fatty acid supplementation improves sow productivity while strengthening offspring intestinal barrier function and mitochondrial integrity, reducing inflammatory injury in piglets. Fang et al. further confirmed that maternal chitosan oligosaccharide supplementation improves milk quality, litter performance, and neonatal immune development while modulating microbial and antioxidant pathways. Together, these studies establish maternal nutrition as a critical upstream regulator of piglet robustness, influencing neonatal survival, gut maturation, and metabolic resilience before post-weaning challenges occur.

## Conclusion

Collectively, the studies in this Research Topic demonstrate that modern swine nutritional strategies are rapidly evolving from single-nutrient approaches toward integrated, multi-targeted systems that combine digestive optimization, microbiome modulation, immune regulation, and maternal programming and collectively highlight that functional nutrients and feed additives can improve gut health and performance; however, their efficacy is highly context-dependent and strongly influenced by environmental hygiene, pathogen load, and production conditions.

Importantly, the data also emphasize that maternal dietary interventions provide a powerful complementary strategy to enhance offspring resilience before weaning, reducing early-life vulnerability and improving long-term performance potential. Across all studies, a consistent conclusion is that gut health in swine is not governed by nutrition alone but by a dynamic interaction between diet, microbiota, host physiology, and environmental exposure.

Future research should therefore prioritize integrative nutritional models that combine maternal and postnatal interventions, identify synergistic additive combinations, and incorporate environmental and management factors into nutritional design. Such a systems-based approach will be essential for reducing reliance on antibiotics, improving animal welfare, and achieving sustainable productivity in modern swine production systems.

